# Epigenetic Biomarkers in Thoracic Aortic Aneurysm, Dissection, and Bicuspid Aortopathy: A Comprehensive Review

**DOI:** 10.3390/biom15040568

**Published:** 2025-04-11

**Authors:** Dimitrios E. Magouliotis, Serge Sicouri, Noah Sicouri, Massimo Baudo, Francesco Cabrucci, Yoshiyuki Yamashita, Basel Ramlawi

**Affiliations:** 1 Department of Cardiac Surgery Research, Lankenau Institute for Medical Research, Main Line Health, Wynnewood, PA 19096, USA; sicouris@mlhs.org (S.S.); massimo.baudo@icloud.com (M.B.); cabruccif@mlhs.org (F.C.); yamashitay@mlhs.org (Y.Y.); ramlawib@mlhs.org (B.R.); 2 Lankenau Medical Center, Wynnewood, PA 19096, USA; noahpierresicouri@gmail.com

**Keywords:** thoracic aortic disease, aortic aneurysm, aortic dissection, epigenetics, DNA methylation, microRNAs, biomarkers

## Abstract

Thoracic aortic disease (TAD) encompasses a spectrum of life-threatening conditions, including thoracic aortic aneurysm (TAA), acute type A aortic dissection (ATAAD), and bicuspid aortic valve (BAV)-associated aortopathy. While genetic mutations are well-documented contributors, emerging evidence highlights epigenetic mechanisms as critical regulators of TAD pathogenesis. This comprehensive review explores the role of epigenetic modifications—DNA methylation, histone modifications, and microRNA (miRNA) regulation—in vascular remodeling, extracellular matrix degradation, and endothelial dysfunction. Aberrant DNA methylation patterns have been implicated in TAA and ATAAD, influencing genes responsible for vascular integrity and inflammation. Histone modifications modulate smooth muscle cell phenotype switching, impacting aneurysm progression. Additionally, dysregulated miRNA expression contributes to endothelial barrier disruption and extracellular matrix remodeling, presenting novel avenues for biomarker discovery. The reversibility of epigenetic modifications offers a promising therapeutic target, with pharmacological agents such as histone deacetylase inhibitors and miRNA-based therapies showing potential in preclinical models. This review underscores the translational potential of epigenetic biomarkers for early disease detection, risk stratification, and precision medicine approaches. Further research is needed to integrate these findings into clinical practice, paving the way for innovative diagnostic and therapeutic strategies in TAD management.

## 1. Introduction

Thoracic aortic disease (TAD) is a collective term for pathological conditions affecting the thoracic aorta, including thoracic aortic aneurysm (TAA), acute aortic syndrome (acute type A aortic dissection—ATAAD; intramural hematoma; penetrating aortic ulcer), and bicuspid aortic valve (BAV)-associated aortopathy. TAA is a chronic disorder characterized by the progressive dilation of the aorta due to structural degeneration of the extracellular matrix (ECM) and vascular smooth muscle cells (VSMCs), which increases the risk of life-threatening complications such as dissection and rupture [1]. Acute type A aortic dissection is a medical emergency in which a tear in the aortic intima allows blood to dissect through the layers of the aortic wall, forming a false lumen, often leading to rapid hemodynamic collapse if left untreated [2]. Additionally, BAV, a congenital condition affecting approximately 1–2% of the population, predisposes individuals to structural abnormalities in the aortic wall, increasing their susceptibility to aneurysm formation and dissection [3].

While TAD has traditionally been associated with genetic syndromes such as Marfan syndrome, Loeys–Dietz syndrome, and Ehlers–Danlos syndrome, the majority of cases are sporadic and not linked to known monogenic mutations [4]. This suggests that non-genetic regulatory mechanisms contribute to disease development and progression. Over the past decade, increasing evidence has highlighted the role of epigenetics in modulating the molecular pathways involved in thoracic aortic disease [5]. Epigenetics refers to heritable yet reversible modifications in gene expression that do not alter the DNA sequence but regulate critical cellular processes, including vascular remodeling, inflammation, and apoptosis. Key epigenetic mechanisms include DNA methylation, histone modifications, and the regulation of gene expression by non-coding RNAs, particularly microRNAs (miRNAs) [6].

DNA methylation, the addition of a methyl- group to cytosine residues within CpG dinucleotides, is a major epigenetic process that influences gene expression by repressing transcription. Abnormal DNA methylation patterns have been reported in patients with TAA, where hypomethylation of ECM-related genes leads to excessive matrix degradation, a key pathological hallmark of aneurysm formation [7]. Furthermore, differentially methylated loci have been identified in ATAAD, with specific epigenetic alterations in genes regulating endothelial integrity and inflammatory responses [8]. Loss of methylation in genes such as TJP2 (zona occludens 2) has been associated with endothelial dysfunction and increased permeability, facilitating aortic wall disruption and dissection progression [9].

In addition to DNA methylation, histone modifications play a pivotal role in controlling gene expression by altering chromatin accessibility. Acetylation and methylation of histone residues regulate the transcriptional activity of genes implicated in vascular homeostasis and smooth muscle cell differentiation. In thoracic aortic aneurysms, reduced histone H3K27 methylation has been linked to the suppression of gap junction proteins, disrupting intercellular communication in VSMCs and promoting aneurysm progression [10]. Moreover, histone deacetylase (HDAC) inhibitors have shown potential in restoring smooth muscle cell function and preventing aneurysm expansion in preclinical models [11].

MicroRNAs (miRNAs) have emerged as crucial regulators of thoracic aortic disease by modulating post-transcriptional gene expression. These small non-coding RNAs bind to target mRNAs and either degrade them or inhibit translation. Several miRNAs have been implicated in TAD, including miR-155-5p and miR-1-3p, which are down-regulated in aortic dissection, leading to endothelial dysfunction and increased vascular permeability [12]. Additionally, miRNAs such as miR-942-5p and miR-5001-3p have been identified as regulators of gap junction proteins in TAA, highlighting their role in VSMC function and aneurysm development [13]. These findings underscore the potential of miRNA-based biomarkers for early disease detection and prognosis.

The growing recognition of epigenetic contributions to thoracic aortic disease has significant implications for both diagnosis and treatment. Unlike genetic mutations, which are fixed and non-modifiable, epigenetic modifications are dynamic and responsive to environmental factors such as diet, smoking, and pharmacological agents [14]. This offers new opportunities for the development of epigenetic-based biomarkers that can enable early detection of high-risk individuals. Blood-based assays measuring circulating miRNAs, DNA methylation patterns, or histone modifications may serve as non-invasive diagnostic tools, allowing for the identification of patients at risk for aneurysm formation or dissection before overt structural changes occur [15].

**Figure 1:**
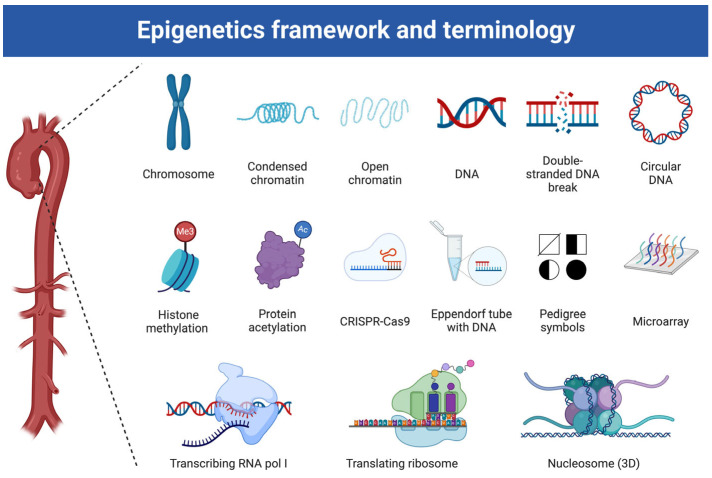
The basic framework and terminology of Epigenetics in Thoracic Aortic Diseases. Created in BioRender. Magouliotis, D. (2025) https://BioRender.com/q25h573 (accessed on 4 March 2025).

Additionally, the reversibility of epigenetic modifications presents promising avenues for therapeutic intervention. Pharmacological agents targeting DNA methyl-transferases (DNMTs), histone deacetylases (HDACs), and miRNA pathways are under investigation for their ability to modify vascular remodeling and prevent disease progression [14]. Figure 1 demonstrates the basic framework of epigenetics in the context of TAD. Experimental studies have shown that HDAC inhibitors can prevent the phenotypic switching of VSMCs, a key contributor to aneurysm growth, while miRNA-based therapies, including miRNA mimics and inhibitors, have demonstrated potential in regulating ECM homeostasis and inflammation [15].

This review aims to provide a comprehensive synthesis of the latest research on epigenetic biomarkers in thoracic aortic disease, focusing on TAA, ATAAD, and BAV-associated aortopathy. In fact, the present review focuses on three major forms of thoracic aortic disease—TAA, ATAAD, and BAV-associated aortopathy—rather than broader vascular diseases like atherosclerosis or calcification. By exploring emerging data on DNA methylation, histone modifications, and miRNA regulation, we seek to highlight the clinical potential of epigenetic biomarkers in improving early diagnosis and targeted treatment strategies. In this study, we decided to follow the structure of a narrative review. While a systematic review would offer methodological rigor, the current literature on epigenetic biomarkers in thoracic aortic disease is highly heterogeneous and limited in scope. Therefore, a comprehensive narrative approach was adopted to integrate experimental, translational, and emerging clinical insights. Furthermore, we discuss the translational challenges associated with integrating epigenetic findings into clinical practice. A deeper understanding of how epigenetic regulation interacts with genetic and environmental factors will pave the way for precision medicine approaches, ultimately improving patient outcomes and reducing the burden of thoracic aortic disease.

## 2. Epigenetic Mechanisms in Thoracic Aortic Disease

Epigenetic modifications regulate gene expression without altering the underlying DNA sequence, thereby influencing vascular homeostasis, smooth muscle cell (SMC) function, endothelial integrity, and extracellular matrix (ECM) remodeling. These modifications include DNA methylation, histone modifications, and non-coding RNAs, particularly microRNAs (miRNAs). Emerging evidence suggests that dysregulated epigenetic processes contribute significantly to the pathogenesis of thoracic aortic aneurysm, acute type A aortic dissection, and bicuspid aortic valve-associated aortopathy. This section explores the role of these mechanisms in thoracic aortic disease and their potential implications for early diagnosis and treatment. Unless otherwise noted, all epigenetic findings refer to human aortic tissue samples.

### 2.1. DNA Methylation and Aortic Wall Integrity

DNA methylation is one of the most well-characterized epigenetic modifications. It involves the addition of a methyl group to cytosine residues in CpG dinucleotides, typically leading to transcriptional repression. Abnormal DNA methylation patterns have been implicated in TAD, particularly in genes regulating ECM stability, vascular SMC behavior, and inflammatory pathways [10,11].

In TAA, studies have identified widespread hypomethylation in genes responsible for ECM integrity, leading to an imbalance between ECM synthesis and degradation. The hypomethylation of genes such as *MMP9* and *MMP2*, which encode matrix metalloproteinases, contributes to increased ECM breakdown and subsequent aortic wall weakening [7]. Similarly, altered methylation of *TGFB2* and *SMAD3*, components of the transforming growth factor-beta (TGF-β) signaling pathway, has been linked to abnormal aortic remodeling and aneurysm formation [16].

Acute type A aortic dissection is associated with epigenetic modifications affecting endothelial integrity and inflammatory responses. Loss of methylation in *TJP2* has been identified in aortic tissue samples from patients with acute type A dissection [17,18]. In fact, such loss of DNA methylation at loci regulating endothelial junction proteins has been shown to compromise endothelial barrier function, increasing vascular permeability and susceptibility to dissection [17]. Furthermore, hypermethylation of anti-inflammatory genes such as *IL10* and *SOCS3* promotes a pro-inflammatory state within the aortic wall, exacerbating disease progression [8].

Bicuspid aortic valve-associated aortopathy also demonstrates unique DNA methylation profiles. Differentially methylated regions have been identified in genes involved in vascular smooth muscle contractility, including *MYH11* and *ACTA2*, which are critical for maintaining aortic wall stability [18]. These findings suggest that DNA methylation alterations contribute to the structural vulnerability observed in BAV patients.

### 2.2. Histone Modifications and Smooth Muscle Cell Dysfunction

Histone modifications, including acetylation, methylation, phosphorylation, and ubiquitination, regulate chromatin structure and transcriptional activity. In thoracic aortic disease, aberrant histone modifications have been implicated in vascular SMC dedifferentiation, ECM remodeling, and endothelial dysfunction [8].

Histone acetylation, mediated by histone acetyltransferases (HATs), promotes gene transcription, whereas histone deacetylation, facilitated by histone deacetylases (HDACs), leads to gene silencing. Increased HDAC activity has been observed in TAA, resulting in the suppression of genes essential for vascular integrity. Specifically, excessive HDAC-mediated deacetylation of *GJA3*, *GJA9*, and *GJC2*, which encode gap junction proteins, disrupts intercellular communication among SMCs, contributing to aneurysm formation [8].

Histone methylation plays a crucial role in the regulation of SMC phenotype switching. Under normal conditions, vascular SMCs maintain a contractile phenotype, ensuring vessel stability. However, in TAA, histone H3K27 methylation is reduced at loci controlling SMC contractile markers, such as *ACTA2* and *MYH11*, leading to SMC dedifferentiation and loss of aortic wall integrity [9]. Inhibition of lysine demethylases (KDMs), which remove histone methylation marks, has been proposed as a potential therapeutic strategy to prevent aneurysm progression.

Additionally, studies have shown that pharmacological inhibition of HDACs can prevent pathological vascular remodeling. HDAC inhibitors such as trichostatin A (TSA) and valproic acid (VPA) have demonstrated protective effects in preclinical models of TAA by preserving the SMC contractile phenotype and reducing ECM degradation [14]. These findings underscore the potential for targeting histone-modifying enzymes in the treatment of thoracic aortic disease.

### 2.3. MicroRNAs: Key Regulators in Aortic Pathology

MicroRNAs (miRNAs) are small non-coding RNAs that regulate gene expression post-transcriptionally by targeting messenger RNA (mRNA) transcripts for degradation or translational inhibition. Dysregulated miRNA expression has been implicated in multiple pathological processes in thoracic aortic disease, including endothelial dysfunction, SMC dedifferentiation, inflammation, and ECM remodeling.

In acute type A aortic dissection, specific miRNAs have been shown to influence endothelial integrity and inflammatory responses. Downregulation of miR-155-5p and miR-1-3p has been associated with impaired endothelial barrier function, leading to increased vascular permeability and susceptibility to dissection [10]. miR-155-5p is measurable in both plasma and tissue samples, with significant downregulation observed in ATAAD patients’ blood, supporting its role as a circulating biomarker. Conversely, upregulation of miR-21 has been shown to enhance fibroblast activation and ECM remodeling, contributing to aneurysm formation [11].

Thoracic aortic aneurysm is characterized by miRNA-mediated regulation of SMC phenotype and ECM stability. miR-942-5p and miR-5001-3p have been identified as key regulators of gap junction proteins, influencing intercellular communication and vascular tone. Additionally, miR-29 family members are known to target multiple ECM genes, including COL1A1, COL3A1, and ELN, thereby modulating aortic wall structure [15].

Bicuspid aortic valve-associated aortopathy shows a distinct miRNA expression profile compared to tricuspid aortic valve-related aneurysms. miR-17-92 cluster dysregulation has been implicated in abnormal SMC proliferation and ECM remodeling in BAV patients [13]. These findings suggest that miRNA-based biomarkers could serve as valuable tools for distinguishing different aortic disease subtypes and guiding personalized therapeutic strategies.

The growing recognition of epigenetic contributions to thoracic aortic disease has significant implications for both diagnosis and treatment. Unlike genetic mutations, which are static, epigenetic modifications are reversible and influenced by environmental factors such as smoking, diet, and pharmacological agents [12]. This highlights the potential for developing epigenetic-based biomarkers and therapies to improve patient outcomes.

## 3. Novel Biomarkers in Specific Aortic Pathologies

Understanding the molecular mechanisms underlying TAD is crucial for early detection, risk stratification, and therapeutic development. Recent advances in transcriptomics and epigenetics have identified several novel biomarkers associated with thoracic aortic aneurysm, acute type A aortic dissection, and bicuspid aortic valve-related aortopathy. These biomarkers, derived from differential gene expression, DNA methylation, histone modifications, and microRNA regulation, offer potential for non-invasive diagnostic assays and targeted therapies. This section provides a detailed overview of novel epigenetic biomarkers in these three major aortic pathologies.

### 3.1. Thoracic Aortic Aneurysm

Thoracic aortic aneurysm is characterized by progressive aortic dilation due to the disruption of ECM integrity, smooth muscle cell dysfunction, and chronic low-grade inflammation. Traditional diagnostic strategies rely on imaging techniques such as computed tomography (CT) angiography and magnetic resonance imaging (MRI); however, these methods detect aneurysms only at later stages. The identification of molecular biomarkers associated with early aneurysm formation could revolutionize screening and risk prediction. Table 1 demonstrates the principal epigenetic mechanisms implicated in TAD biology.

### 3.2. Differentially Expressed Genes and DNA Methylation in TAA

Recent transcriptomic studies have identified numerous differentially expressed genes (DEGs) involved in ECM remodeling, vascular inflammation, and endothelial dysfunction in TAA. For example, genes such as *MMP9* and *MMP2*, encoding matrix metalloproteinases, are significantly upregulated in aneurysmal aortic tissue, contributing to ECM degradation and aneurysm expansion [19]. MMP2 and MMP9 contribute directly to ECM degradation and are also detectable in circulation, where elevated levels have been proposed as biomarkers for TAA and ATAAD [19].

Epigenome-wide association studies have revealed that DNA methylation alterations also play a key role in TAA progression. Studies have shown that *TGFB2* and *SMAD3*, key regulators of TGF-β signaling, exhibit hypomethylation in aneurysmal tissue, leading to aberrant activation of ECM remodeling pathways [20]. Additionally, hypermethylation of *ACTA2* and *MYH11*, two genes essential for SMC contractility, has been associated with aortic wall weakening and aneurysm development [18].

SIRT6: Recent studies have identified SIRT6 as an epigenetic repressor that protects against TAA by inhibiting vascular inflammation and cellular senescence. The loss of SIRT6 function has been associated with increased susceptibility to aneurysm formation, suggesting its potential as a therapeutic target [21].

Hox Genes: Altered DNA methylation patterns in Hox gene clusters have been implicated in TAA pathogenesis. Hypermethylation of specific Hox genes correlates with their downregulation, contributing to vascular smooth muscle cell (VSMC) dysfunction and aortic wall instability. These epigenetic changes may serve as biomarkers for early disease detection [22].

### 3.3. MicroRNAs and Histone Modifications in TAA

While several epigenetic markers identified in TAA studies derive from tissue-based analyses, circulating forms—particularly miRNAs like miR-29 and miR-21—have also been detected in plasma, raising their potential for non-invasive diagnostics. MicroRNAs (miRNAs) regulate gene expression post-transcriptionally and have emerged as key modulators of TAA pathology. Downregulation of miR-29 family members, particularly miR-29a and miR-29b, has been linked to increased collagen and elastin degradation, exacerbating aneurysm expansion [11]. Similarly, miR-21 has been identified as a critical regulator of fibroblast activation and ECM turnover in aneurysmal tissue [15].

Histone modifications further contribute to TAA progression by altering chromatin accessibility. Reduced *H3K27* trimethylation at loci encoding ECM proteins has been shown to promote aneurysm growth by increasing the expression of pro-inflammatory genes [8]. Histone deacetylase (HDAC) inhibitors, such as valproic acid, have demonstrated potential in reversing these epigenetic changes and stabilizing aneurysmal growth in preclinical models [14].

### 3.4. Acute Type A Aortic Dissection (ATAAD)

Acute type A aortic dissection is a life-threatening condition requiring immediate surgical intervention. The rapid onset and catastrophic nature of dissection highlight the urgent need for biomarkers that can identify high-risk individuals prior to clinical presentation. Epigenetic biomarkers in ATAAD primarily involve genes regulating endothelial integrity, inflammation, and oxidative stress.

Several inflammatory biomarkers have been linked to ATAAD. Elevated plasma levels of interleukin-10 (IL-10) have been observed in ATAAD patients compared to those with other cardiovascular conditions [23]. This cytokine’s anti-inflammatory properties and its significant increase during dissection events suggest its utility as a diagnostic biomarker. Similarly, C-reactive protein (CRP), high-sensitive troponin T (hs-TnT), interleukin 6 (IL-6), and plasminogen activator inhibitor 1 (PAI1) have been associated with increased mortality in ATAAD patients, making its monitoring valuable for risk stratification and management decisions [23].

Endothelial cell dysfunction plays a pivotal role in the pathogenesis of ATAAD. The disruption of tight junctions and increased vascular permeability predispose the aortic wall to mechanical stress and rupture. DNA methylation analyses have revealed hypermethylation of genes encoding endothelial barrier proteins, including TJP2 (zona occludens 2), leading to decreased expression and compromised endothelial integrity [17]. Additionally, epigenetic regulation of inflammatory mediators is critical in dissection pathology. Hypomethylation of IL6 and TNF-α promoters has been linked to a pro-inflammatory state within the aortic wall, exacerbating oxidative stress and ECM degradation [8]. The upregulation of IL-6 and TNF-α in ATAAD has been linked to hypomethylation at their promoter regions, indicating that inflammatory activation may be epigenetically regulated in the acute disease setting [8]. To date, no published studies have reported histone acetylation or deacetylation events specifically associated with ATAAD. This represents a significant gap in the literature and an opportunity for future investigation.

MicroRNAs have also been identified as potential biomarkers for ATAAD. miR-155-5p, a key regulator of endothelial inflammation, is significantly downregulated in dissected aortic tissue, resulting in increased vascular permeability and leukocyte infiltration [10]. Certain miRNAs such as miR-155-5p and inflammatory markers like IL-10 have been detected in plasma samples from ATAAD patients, supporting their feasibility as circulating biomarkers. However, others—like histone modifications or TJP2 methylation—currently require tissue-based profiling and thus remain investigational. In fact, miR-155-5p is measurable in both plasma and tissue samples, with significant downregulation observed in ATAAD patients’ blood, supporting its role as a circulating biomarker [10]. In contrast, miR-21 is upregulated in ATAAD patients and has been implicated in excessive fibroblast proliferation and ECM remodeling, further destabilizing the aortic wall [11]. Histone modifications have also been implicated in ATAAD. Increased H3K4 methylation at inflammatory gene promoters has been associated with enhanced expression of MMP9, accelerating ECM degradation and increasing susceptibility to dissection [9].

### 3.5. Bicuspid Aortic Valve (BAV)-Associated Aortopathy

BAV-associated aortopathy represents a distinct form of thoracic aortic disease, characterized by early-onset aneurysm formation and increased risk of dissection compared to tricuspid aortic valve (TAV) patients. Unique epigenetic signatures distinguish BAV aortopathy from TAV-related aneurysms. The clinical translation of epigenetic biomarkers in BAV aortopathy remains in early stages. Most findings, including NOTCH1 hypermethylation and miRNA dysregulation, are based on aortic wall samples; future studies should evaluate whether these changes are reflected in peripheral blood.

Studies have shown that differential DNA methylation patterns exist between BAV and TAV patients. Hypermethylation of NOTCH1, a gene crucial for valve development, has been observed in BAV-associated aortopathy, potentially contributing to structural abnormalities and altered aortic wall composition [14]. Transcriptomic analyses have revealed increased expression of P2RX4 and KDR, genes involved in endothelial signaling, in BAV aortopathy patients. These findings suggest a unique molecular trajectory compared to sporadic TAA [7]. Distinct miRNA profiles differentiate BAV from TAV aneurysms. Dysregulation of the miR-17-92 cluster has been associated with abnormal SMC proliferation and ECM remodeling in BAV patients, potentially driving early aneurysm formation [13]. Additionally, miR-296-5p and miR-6132 regulate key pathways involved in aortic wall stability, making them promising candidates for future biomarker development. Although DNA methylation and miRNA signatures have been extensively profiled in BAV-associated aortopathy, histone acetylation and deacetylation remain underexplored in this disease subtype.

### 3.6. Emerging Biomarker Candidates

The search for robust biomarkers in thoracic aortic disease (TAD) has expanded beyond traditional pathways to include novel proteins, non-coding RNAs, and complex gene networks. These emerging candidates hold promise for enhancing diagnostic precision and risk stratification (Table 2).

One promising category of biomarkers involves gap junction proteins (GJPs), which facilitate intercellular communication and maintain aortic wall integrity [24]. Dysregulation of these proteins disrupts cellular cohesion and promotes vascular dysfunction. Computational analyses have identified eight downregulated GJPs in TAD, including GJA3, GJA9, GJC2, and GJD3 [24]. These proteins are implicated in electrical coupling and signal transduction, which are essential for smooth muscle cell coordination. Additionally, regulatory miRNAs such as hsa-miR-942-5p and hsa-miR-6867-3p have been found to modulate GJP expression, representing potential therapeutic targets.

**Figure 2:**
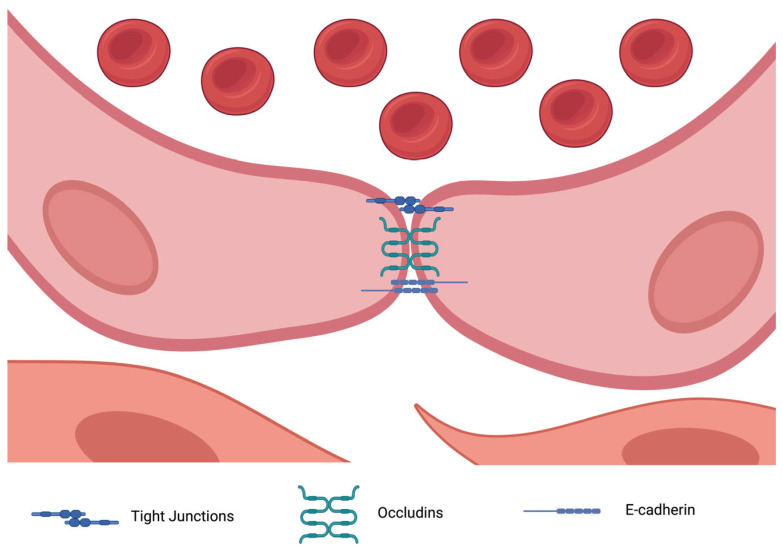
The main intercellular junctions implicated in thoracic aortic diseases. Created in BioRender. Magouliotis, D. (2025) https://BioRender.com/s40i249 (accessed on 4 March 2025).

Tight junction proteins (TJPs) also play a crucial role in endothelial barrier integrity by preventing vascular leakage (Figure 2). Disruption of these junctions contributes to TAD pathogenesis. For example, zona occludens proteins ZO-1 and ZO-2 (encoded by TJP2) have been found to be underexpressed in thoracic aortic dissection (TAD), leading to endothelial dysfunction and exacerbating wall instability [25]. Additionally, miRNAs such as hsa-miR-155-5p have been linked to the dysregulation of ZO-2, further compromising endothelial integrity. Proteins such as CLDN5 and CLDN11 (claudins) are also downregulated in TAD, correlating with increased vascular permeability and inflammation [26].

Smooth muscle cell dysfunction is another hallmark of TAD, and novel markers have been identified in this domain. MYOCD (myocardin), a master regulator of SMC differentiation, has been found to be epigenetically silenced in TAD, leading to a shift from a contractile to a synthetic phenotype, which contributes to aneurysm formation [27]. CNN1 (calponin 1), another SMC-specific protein, exhibits reduced expression in aneurysmal tissue, further highlighting the role of epigenetic regulation in SMC dysfunction [28].

Inflammatory pathways also contribute significantly to TAD progression, and epigenetic modulation of inflammatory mediators has revealed new potential biomarkers. Promoter hypomethylation of IL-6 and TNF-α leads to their overexpression, perpetuating an inflammatory milieu in the aortic wall [29]. Additionally, NOX4 (NADPH oxidase 4), an oxidative stress marker regulated by miR-25-3p, has been found to be overexpressed in TAD, contributing to the production of reactive oxygen species (ROS) and subsequent aortic wall damage [30].

Beyond well-established miRNAs, several emerging non-coding RNAs have been implicated in TAD. miR-155, a pro-inflammatory miRNA, has been shown to upregulate matrix metalloproteinases, leading to excessive ECM degradation [31]. Additionally, the long non-coding RNA (lncRNA) ANRIL has been associated with inflammation and cell cycle regulation in vascular smooth muscle cells, providing further insight into the complex regulatory mechanisms underlying TAD progression [32].

### 3.7. Clinical Translation and Challenges

**Figure 3:**
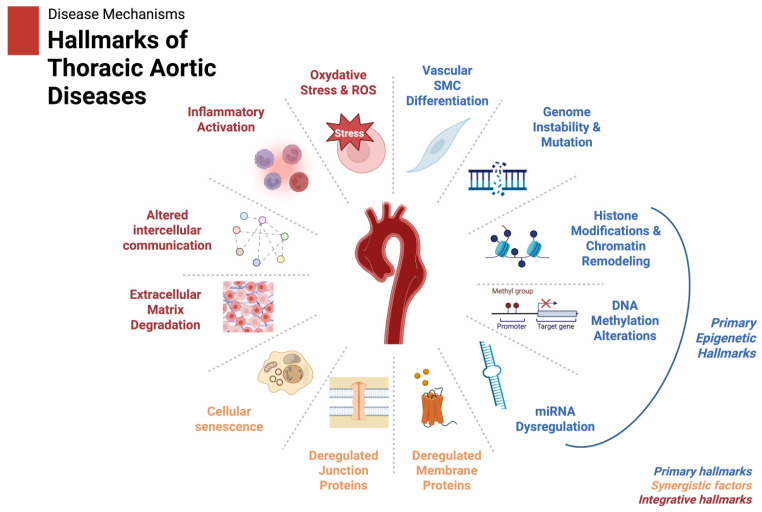
The hallmarks of thoracic aortic diseases, including those influenced by epigenetic regulation (e.g., miRNA dysregulation, histone modification, and DNA methylation), are illustrated. Integrative factors like inflammation and ECM remodeling are shown to reflect the downstream effects of these epigenetic changes. Created in BioRender. Magouliotis, D. (2025) https://BioRender.com/x15e086 (accessed on 4 March 2025).

The discovery of epigenetic biomarkers in TAD holds immense potential for improving diagnosis, prognosis, and therapeutic interventions. Based on these findings we propose a scheme of the hallmarks of thoracic aortic diseases as shown in Figure 3. However, translating these findings into clinical practice poses several significant challenges. One of the primary obstacles is biomarker validation. Many studies to date have relied on small sample sizes, often with limited diversity in terms of ethnicity, age, or comorbidities [33,34,35]. The heterogeneity in TAD etiologies—ranging from syndromic conditions such as Marfan syndrome to sporadic cases—further complicates biomarker standardization. Therefore, robust multicenter studies with large, well-characterized cohorts are essential to establish the clinical utility and reproducibility of epigenetic markers.

Non-invasive detection techniques also present a major challenge. Although epigenetic biomarkers show promise for early diagnostics, translating tissue-based findings to peripheral blood or other biofluids remains difficult. Many biomarkers identified in aortic tissue, such as miRNAs or DNA methylation changes, may not be easily detectable in circulation or may exhibit variable expression levels [36]. Developing highly sensitive and specific assays, such as digital PCR or next-generation sequencing platforms, will be crucial for enabling the widespread use of circulating biomarkers in clinical settings.

Another critical aspect of clinical implementation is the integration of epigenetic biomarkers with existing clinical tools. While imaging techniques such as CT and MRI remain the gold standard for assessing aortic size and structural abnormalities, biomarkers could complement these modalities by improving early detection and risk stratification. Hybrid approaches that combine imaging data with biomarker profiles will require rigorous testing and optimization to ensure their relevance in clinical decision-making.

Beyond scientific and technical challenges, regulatory and economic considerations must also be addressed. The development and approval of epigenetic biomarkers require navigating complex regulatory pathways [37]. Biomarkers must demonstrate high sensitivity, specificity, and clinical utility in regulatory-grade trials before they can be approved by agencies such as the FDA or EMA [38]. Additionally, cost-effectiveness analyses are necessary to justify their implementation in routine practice, particularly in healthcare systems with limited resources.

Ethical and privacy concerns further complicate the clinical adoption of epigenetic biomarkers. Like genetic data, epigenetic information carries significant ethical implications, particularly regarding patient privacy and data sharing. Epigenetic changes can reflect environmental exposures and lifestyle factors, raising potential concerns about stigmatization or discrimination [39]. Establishing clear guidelines for the ethical use and secure storage of epigenetic data will be imperative to ensure patient confidentiality and public trust.

### 3.8. Biological Insights and Pathophysiological Implications

Epigenetic changes in TAD provide mechanistic insights into disease pathogenesis, highlighting key molecular pathways that contribute to aortic wall weakening. Aberrant methylation of genes such as MMP9 and TGFBR1 underscores the role of dysregulated ECM remodeling and transforming growth factor-beta (TGF-β) signaling in disease progression [38]. Similarly, the downregulation of miRNAs such as miR-29 and miR-145 highlights their importance in maintaining vascular integrity by modulating SMC phenotype and ECM homeostasis [40].

The identification of epigenetic changes in gap and tight junction proteins, such as GJA3 and ZO-2, further emphasizes the role of endothelial cell dysfunction in disease progression. These findings align with the broader understanding that vascular inflammation, oxidative stress, and biomechanical stressors act synergistically to destabilize the aortic wall. The dynamic and reversible nature of epigenetic changes makes them particularly attractive as biomarkers and therapeutic targets, offering opportunities to monitor disease progression and intervene before catastrophic events such as aortic dissection occur.

### 3.9. Future Directions

To fully realize the potential of epigenetic biomarkers in TAD, a multifaceted approach combining technological innovation, rigorous research, and interdisciplinary collaboration is required. Future research should focus on multi-omics integration, combining epigenetic data with genomics, transcriptomics, proteomics, and metabolomics. This comprehensive approach will enable the identification of robust biomarker panels that reflect the complex interplay of genetic and environmental factors in TAD pathogenesis. Advanced bioinformatics and machine learning models will be instrumental in synthesizing these data layers to uncover novel disease pathways and predictive signatures.

Longitudinal studies are also necessary to track the temporal dynamics of epigenetic changes in TAD. Most existing studies provide only a snapshot of these alterations at a single time point. By conducting long-term studies, researchers can identify biomarkers that predict disease onset, progression, or response to therapy. Such studies could also help differentiate between stable aneurysms and those at imminent risk of dissection.

Finally, the field must advance toward personalized medicine. Epigenetic biomarkers offer a promising pathway toward individualized treatment strategies, enabling clinicians to stratify patients based on their unique epigenetic profiles. This could guide decisions regarding the timing of surgical interventions, pharmacological treatments, or lifestyle modifications. For instance, patients with specific miRNA dysregulation might benefit from targeted RNA therapeutics, while those with aberrant DNA methylation patterns could be candidates for demethylating agents.

With continued research and technological advancements, epigenetic biomarkers could become an integral component of precision medicine in TAD, ultimately improving patient outcomes through earlier diagnosis, better risk stratification, and targeted therapeutic interventions.

## 4. Conclusions

The identification of novel biomarkers at the epigenetic level offers promising avenues for early diagnosis, risk stratification, and therapeutic intervention in thoracic aortic disease. DNA methylation patterns, histone modifications, and miRNA signatures provide insights into the molecular mechanisms underlying TAA, ATAAD, and BAV-associated aortopathy. Future studies should focus on translating these biomarkers into non-invasive blood-based assays for early detection and targeted epigenetic therapies aimed at preventing disease progression.

## Figures and Tables

**Table 1:** The principal epigenetic mechanisms implicated in TAD biology.

Epigenetic Mechanisms	Key Targets	Effect on TAD
DNA Methylation	MMP9, TGFB2, SMAD3	Regulates ECM Degradation and Endothelial Integrity
Histone Modification	H3K27, HDACs	Affects Smooth Muscle Cell Phenotype and Vascular Inflammation
MicroRNA (miRNA) Regulation	miR-29, miR-21, miR-155-5P	Controls Post-transcriptional Gene Expression in Vascular Remodeling

**Table 2:** Key epigenetic biomarkers and their implication in thoracic aortic diseases.

Aortic Disease Type	Biomarker Type	Biomarker	Mechanism	References
TAA	Epigenetic Regulator	SIRT6 (Histone deacetylase)	Protects against aneurysm progression by reducing inflammation and cellular senescence.	Cardus et al., 2013 [21]
TAA	Histone Modification	H3K27 Trimethylation	Loss of this histone mark leads to pro-inflammatory gene activation and aortic weakening.	Boileau et al., 2018 [10]
ATAAD	Inflammatory Cytokine	Interleukin-10 (IL-10)	Elevated plasma levels correlate with higher inflammation and disease severity.	Forrer et al., 2021 [23]
ATAAD	Epigenetic Modification	TJP2 (Zona Occludens 2) Methylation	Hypermethylation results in endothelial barrier dysfunction, increasing dissection risk.	Pan et al., 2017 [18]
ATAAD	MicroRNA	miR-155-5p	Downregulation leads to endothelial dysfunction and increased vascular permeability.	Boileau et al., 2018 [10]
BAV-Associated Aortopathy	Genetic/Epigenetic	NOTCH1 DNA Methylation	Hypermethylation affects valve development, increasing aortic instability.	Pan et al., 2017 [18]
BAV-Associated Aortopathy	MicroRNA	miR-17-92 Cluster	Dysregulation affects smooth muscle cell proliferation and ECM remodeling.	Portelli et al., 2018 [13]

Abbreviations: TAA = thoracic aortic aneurysm; ATAAD = acute type A aortic dissection; BAV = bicuspid aortic valve.

## Data Availability

The data that support the findings of this study are available from the corresponding author, upon reasonable request.
